# New O3-Type Layer-Structured Na_0.80_[Fe_0.40_Co_0.40_Ti_0.20_]O_2_ Cathode Material for Rechargeable Sodium-Ion Batteries

**DOI:** 10.3390/ma14092363

**Published:** 2021-05-01

**Authors:** Daniel A. Anang, Deu S. Bhange, Basit Ali, Kyung-Wan Nam

**Affiliations:** 1 Department of Energy and Materials Engineering, Dongguk University-Seoul, Seoul 04620, Korea; taadjah@yahoo.com (D.A.A.); basitalikhan077@gmail.com (B.A.); 2 Department of Chemical Engineering, Kwame Nkrumah University of Science and Technology, PMB, Kumasi, Ghana; 3 Department of Chemistry, Shivaji University, Kolhapur 416004, India; bhangeds@yahoo.co.in

**Keywords:** Na-ion battery, layered structure, cathode, in situ XRD, XANES

## Abstract

Herein, we formulated a new O3-type layered Na_0.80_[Fe_0.40_Co_0.40_Ti_0.20_]O_2_ (NFCTO) cathode material for sodium-ion batteries (SIBs) using a double-substitution concept of Co in the parent NaFe_0.5_Co_0.5_O_2_, having the general formula Na_1-x_[Fe_0.5–x/2_Co_0.5–x/2_M^4+^_x_]O_2_ (M^4+^ = tetravalent ions). The NFCTO electrode delivers a first discharge capacity of 108 mAhg^−1^ with 80% discharge capacity retention after 50 cycles. Notably, the first charge–discharge profile shows asymmetric yet reversible redox reactions. Such asymmetric redox reactions and electrochemical properties of the NFCTO electrode were correlated with the phase transition behavior and charge compensation reaction using synchrotron-based in situ XRD and ex situ X-ray absorption spectroscopy. This study provides an exciting opportunity to explore the interplay between the rich chemistry of Na_1–x_[Fe_0.5–x/2_Co_0.5–x/2_M^4+^_x_]O_2_ and sodium storage properties, which may lead to the development of new cathode materials for SIBs.

## 1. Introduction

Thanks to their high energy and power density and long cycle life, lithium-ion batteries (LIBs) are currently the most marketable rechargeable batteries for mobile electronics applications [1]. During the past decade, LIBs have been enormously successful in the transportation industry, as they are perceived as a better option in mitigating environmental pollution created by vehicles using conventional combustion engines [2]. Despite this success, the uneven distribution and nonabundant nature of Li resources in the earth crust make LIB technology unsustainable in large-scale energy storage systems (ESSs) in the long term [3,4]. Sodium-ion batteries (SIBs) offer an attractive alternative to LIBs in such large-scale ESSs thanks to their similar intercalation chemistry to LIBs, low cost, and high material abundance [1].

Recently, various sodium (Na) layered oxide materials have been discovered, and their electrochemical properties have been critically examined [4,5,6]. In particular, the α-NaFeO_2_ material reported by Yabuuchi et al. exhibited a flat voltage plateau at 3.3 V vs. Na^+^/Na with 80 mAhg^−1^ reversible capacity coupled with reasonable stability up to 30 cycles [7]. However, when the operating voltage was increased above 3.4 V, significant capacity fading was observed. Structural studies revealed Fe ion migration from the transition metal (TM) layer to the Na layer, leading to irreversible structural change during cycling at higher voltages. Subsequently, Yoshida et al. designed NaFe_0.5_Co_0.5_O_2_ material by substituting half of Fe by Co and obtained improved electrochemical performance over NaFeO_2_ [8]. This cathode material extended the discharge capacity to 160 mAhg^−1^ with ~85 % discharge capacity retention after five cycles and showed high rate (30C) capability. Despite these performances, it was concluded that the Co content must be minimized to successfully implement large-scale practical SIBs, due to the lack of earth abundance of Co. Moreover, Co is a lethally toxic element, and the exposure to living cells can be hazardous [9]. Yabuuchi et al. demonstrated the use of all-earth-abundant elements in P2-type Na_x_[Fe_1/2_Mn_1/2_]O_2_ for practical application in SIBs. In particular, the Na_2/3_[Fe_1/2_Mn_1/2_]O_2_ material produced a high reversible capacity of ~190 mAhg^−1^ when cycled at 13 mAg^−1^ (i.e., 1/20 C rate) within a potential window of 1.5–4.2V. The electrode showed relatively stable cycling performance up to 30 cycles, accessing both Fe^3+^/Fe^4+^ and Mn^3+^/Mn^4+^ redox centers with a single electron redox process [10].

Adopting a similar strategy, we attempted to minimize both Co (due to scarcity and toxicity issues) and Fe (due to inherent structural problems) and formulated a new chemistry that utilizes earth-abundant and nontoxic Ti while maintaining good electrochemical performance. Herein, we adopted the double-substitution concept of Fe^3+^ (high spin, ionic radius = 0.645 Å), and Co^3+^ (high spin, ionic radius = 0.610 Å) in NaFe_0.5_Co_0.5_O_2_ with Ti^4+^ (ionic radius = 0.605 Å), and designed a new O3-type layered Na_0.80_Fe_0.40_Co_0.40_Ti_0.20_O_2_ (denoted here as NFCTO) material. The NFCTO cathode delivers a first discharge capacity of 108 mAhg^−1^ with 80% discharge capacity retention after 50 cycles. Notably, the first charge–discharge profile shows asymmetric yet reversible redox reactions. We also investigated such asymmetric redox reactions and electrochemical properties in terms of the phase transition behavior and charge compensation mechanism of the NFCTO electrode using synchrotron-based in situ XRD and ex situ X-ray absorption spectroscopy.

## 2. Materials and Methods

The sample was synthesized by a solid-state process. Stoichiometric amounts of Na_2_CO_3_, Fe_2_O_3_, Co_3_O_4_, and TiO_2_ powders (bought from Aldrich Chemical Company) were thoroughly mixed in an agate mortar to produce a fine powder, to which 5% excess Na_2_CO_3_ was added to compensate for possible Na vaporization at high temperatures. The mixed powder was pelletized and heated in a tubular furnace with the aid of an alumina boat. Heating was conducted at 5 °C/min from room temperature (RT) to 1000 °C, and kept at this temperature for 15 h. Afterward, the product was left to cool to RT naturally.

The crystal structure of the sample was first analyzed using Rigaku Ultima IV, with a CuK_α_ source and a diffracted beam monochromator. With a scan rate of 2°/min and a step size of 0.02°, powder patterns were collected in the 2θ range of (10–80)°. Morphological features and chemical compositional analyses of the samples were probed using Field Emission Scanning Electron Microscopy (FE-SEM-Inspect F) equipped with energy-dispersive X-ray spectroscopy (EDS). High-Resolution Powder Diffraction (HRPD) was performed at the 9B synchrotron beamline of the Pohang Acceleration Laboratory (PAL, Korea) using monochromated X-rays (λ = 1.5183 Å). In-situ XRD data were collected in the first cycle at the PAL, 6D-UNIST beamline, using a synchrotron source with λ = 0.5949 Å. The in situ cells were cycled using a Wonatech (3000K8) cycler at a constant current rate of 0.1C in the voltage range of 2.0–4.0 V vs. Na^+^/Na (1C = 202.5 mAhg^−1^). Ex situ X-ray absorption spectroscopy spectra of the NFCTO electrodes were obtained at the pristine, half-charged (3.3 V), fully charged (4.0 V), half-discharged (3.2 V), and fully discharged (2.0 V) states at the 7D, 8C, and 10C beamlines of the Pohang Accelerator Laboratory (PAL). Fe, Co, and Ti K-edge spectra were collected in transmission mode, and processed based on the standard data reduction procedure, using the Athena program [11]. Fe and Co K-edge extended X-ray absorption fine structure (EXAFS) spectra were fitted using the Arthemis software for obtaining the quantitative structural information. The extracted EXAFS signal, χ(k), was weighted by *k*^2^ and then Fourier-transformed in *k*-range of 3.0 ~ 11.0 Å^−1^ using a Hanning window function to obtain the magnitude plots in R-space (Å). The Fourier-transformed peaks were not phase corrected such that the actual bond lengths are approximately 0.2–0.4 Å longer. The EXAFS fitting analysis was performed for the first two shells (R range: 1.0 ~3.0 Å) using the single scattering path of TM-O_6_ and TM-TM_6_ (TM = transition metals).

For electrochemical tests, the positive electrode slurry was formulated with 80 wt.% active material, 10 wt.% carbon black, and 10 wt.% polyvinylidene fluoride (PVDF) binder in N-Methyl-2-pyrrolidone (NMP). This slurry was then cast on Al foil, and vacuum dried at 120 °C overnight. The loading amounts of the positive electrodes ranged from 2.0 to 3.0 mg/cm^2^. Coin type (R2032) cells using 12 mm diameter circular disk electrodes were assembled in an argon-filled glovebox. Na metal was used as a negative electrode. The 1 M NaPF_6_ in ethylene carbonate (EC)/propylene carbonate (PC)/dimethyl carbonate (DMC) (1:1:1 vol.%) was used as an electrolyte with a glass fiber membrane as a separator. Galvanostatic charge–discharge experiments were conducted with a Wonatech cycler at RT.

## 3. Results and Discussion

### 3.1. NFCTO Structure

**Figure 1:**
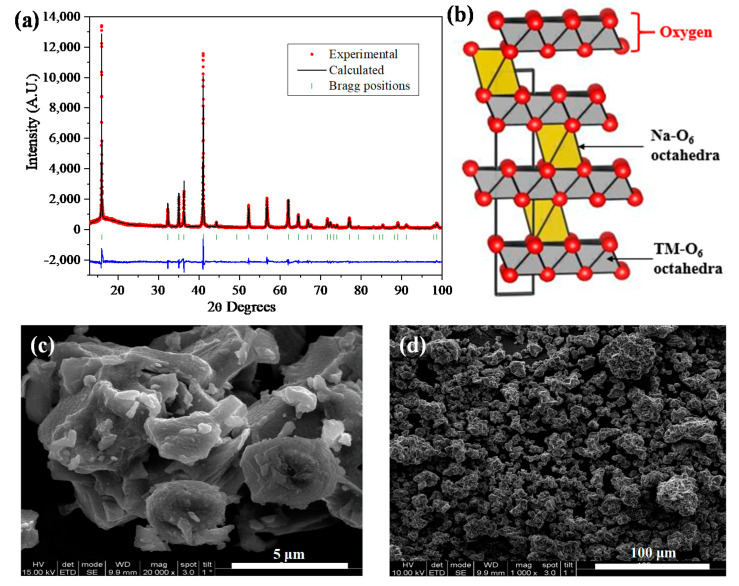
(**a**) Rietveld refinement plot and NFCTO HRPD patterns, (**b**) polyhedral representation of the O3 structure of Na_0.8_Fe_0.4_Co_0.4_Ti_0.2_O_2_: yellow octahedra represent sodium, while gray represents Fe/Co/Ti octahedra. SEM images of NFCTO at (**c**) higher and (**d**) lower magnifications.

The NFCTO material was prepared by a traditional solid-state method. Crystal structure and phase purity with different calcination temperatures of 800–1000 °C were identified by laboratory XRD, as shown in Supplementary Materials Figure S1 of the Supplementary Information (SI). The phase pure O3-type layered structure was obtained at 1000 °C, evidenced by the disappearance of the weak impurity reflections at 19.1, 31.4, and 38.1 2θ degrees. Hence, further investigations were focused on the sample heated at 1000 °C. To quantify the structural details, Rietveld refinement was performed on the high-resolution powder diffraction (HRPD) data (collected using synchrotron source) of the NFCTO sample prepared at 1000 °C (Figure 1a). The calculated XRD patterns (black line in Figure 1a) are in good agreement with the experimentally observed XRD patterns (red circles Figure 1a), confirming the formation of phase pure O3-type layered structure (space group: R$\bar{3}$m), analogous to the α-NaFeO_2_ structure. Supplementary Materials Table S1 of the SI lists the refinement parameters and atomic coordinates. 

Figure 1b displays the polyhedral representation of the NFCTO structure. The structure of NFCTO is made up of packing of transition metal layers (‘ab’ plane) along the ‘c’ axis. Between the transition metal layers, sodium ions are sandwiched with octahedral coordination. The fractional substitution of trivalent transition metal ions (Fe^3+^/Co^3+^) with tetravalent Ti^4+^ ion produced sodium ion deficiencies, which facilitate the rapid two-dimensional diffusion of Na^+^. The final refined lattice parameters of the hexagonal unit cell were *a* = *b* = 2.9475 (1) Å, and *c* = 16.3621 (2) Å. These cell constants (*a* and *b* values) are closely similar to those of the Na_0.8_Ni_0.2_Fe_0.2_Co_0.2_Mn_0.2_Ti_0.2_O_2_ material reported earlier by our group, suggesting only slight changes within the transition metal layer, despite the considerable change in composition [12]. However, it is worth noting that the relatively larger *c* lattice constant (16.3621 (2) Å) is attributed to the Na ion vacancies in the sandwiched sodium ion layers. The TM-O distance was found to be 1.997 (1) Å while the Na-O distance was 2.393 (2)Å, and these bond distances are in accordance with the ionic radii of the corresponding ions.

Figure 1c,d illustrates the SEM images of the NFCTO particles at different magnifications, and the primary particle size was found to be ~4 μm. The corresponding characteristic EDS spectrum shown in Supplementary Materials Figure S2 of the SI confirms the presence of all expected elements (Na, Fe, Co, Ti, and O). The insert of the EDS spectrum (Supplementary Materials Figure S2 of the SI) shows the corresponding elemental weight and atomic %, which agrees well with the target chemical composition of the Na_0.8_Fe_0.4_Co_0.4_Ti_0.2_O_2_ material.

### 3.2. Electrochemical Performance

**Figure 2:**
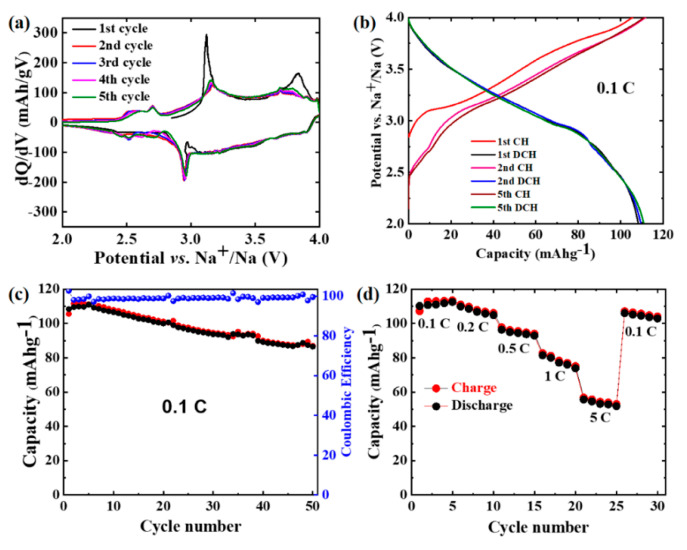
Electrochemical performance of NFCTO electrode, (**a**) dQ/dV plot for cycles 1–5 at 0.1C, (**b**) charge/discharge curves at 0.1C for the 1st, 2nd, and 5th cycles, (**c**) charge/discharge capacities at 0.1C between 2 and 4 V vs. Na^+^/Na, and (**d**) rate performance at various current rates.

The electrochemical performance of the NFCTO electrode was evaluated using galvanostatic charge/discharge measurements between 2–4 V vs. *Na^+^/Na*. Figure 2a illustrates the differential capacity plots (dQ/dV vs. V) cycled at 0.1C for the first five cycles. In the first cycle, a pair of oxidation peaks at ∼3.12 and 3.84 V in the anodic scan and a reduction peak at ∼2.95 V in the cathodic scan are observed. In subsequent cycles, all redox peaks remained stable, although the intensity of the anodic peaks reduced after the first cycle, suggesting highly reversible redox reactions. We assign these redox peaks to the synergistic effect of Fe^4+/3+^and Co^4+/3+/2+^ redox couples, as will be discussed later in the X-ray absorption spectroscopy section [3].

Figure 2b shows the charge/discharge profiles for the 1st, 2nd, and 5th cycles of the electrode at 0.1C. During the first charge, a voltage plateau is observed between 3.10 and 3.15 V, followed by a sloping voltage profile above 3.15 V to the end of the charge. During discharge, a sloping voltage profile is observed throughout discharge, which implies an asymmetric redox reaction mechanism during the first cycle. We later discuss this asymmetric nature of redox reaction in the following in situ XRD and X-ray absorption spectroscopy sections. The charge and discharge capacities obtained in the first cycle are 105.53 and 108.31 mAhg^−1^, respectively. Cycles 2–5 show the same sloping voltage profiles during charge and discharge and exhibit similar capacities of 111 and 110.9 mAhg^−1^, respectively, suggesting robust and highly reversible redox reactions enabled by facile Na extraction/insertion. The electrode shows good cycling stability (Figure 2c), delivering stable charge and discharge capacities of ∼87 and 86.5 mAhg^−1^, respectively, after 50 cycles at 0.1C, which translates into 83% capacity retention. A 103% coulombic efficiency (CE) was observed in the first cycle, due to the insertion of extra Na ions originated from the Na deficient nature of the electrode. The CE stabilized around 100% up to 50 cycles in subsequent cycles, indicating highly reversible reactions during Na^+^ insertion/extraction. 

Figure 2d shows the rate test results of the NFCTO electrode. The electrode exhibits considerable capacities even at high 1.0C and 5.0C rates with respect to the low rate capacity observed at 0.1C. For example, the electrode delivered 81 (at 1.0C) and 56 (at 5.0C) mAhg^−1^, which corresponds to 74 and 51% of the 0.1C capacity, respectively. This result suggests that the NFCTO electrode can be applied to applications requiring high-rate capability. After experiencing these high currents, the electrode again recovered high and stable capacities, when the current rate was restored to its initial value (0.1C). These good electrochemical characteristics indicate that the NFCTO electrode is a promising candidate for practical applications in SIBs.

### 3.3. In Situ XRD Analysis

**Figure 3:**
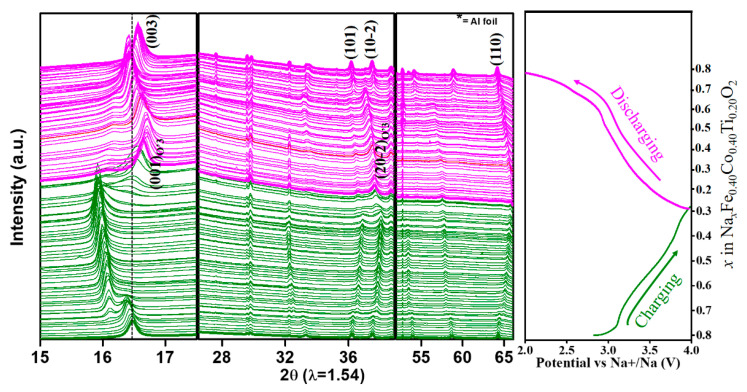
In situ XRD patterns for NFCTO electrode during the first charge and discharge at 0.1C in the voltage range of 2.0–4.0 V vs. *Na^+^/Na*.

We further investigated the origin of the excellent reversibility and asymmetric redox reaction mechanism of the NFCTO electrode using synchrotron-based in situ XRD studies during the first charge and discharge at a 0.1C rate (Figure 3). At the beginning of Na extraction from the Na_x_Fe_0.40_Co_0.40_Ti_0.20_O_2_(x = 0.8) electrode, the (003) reflection of the pristine O3-phase marginally shifted to lower 2θ up to x = 0.75, indicating a typical expansion of the interlayer distance. Simultaneously, the (101) and (10-2) reflections shifted to higher 2θ values suggesting the shrinkage of the transition metal layer (i.e., ‘ab’ plane), due to the oxidation of Co^3+^ and Fe^3+^.

For Na extraction in the range (0.68 ≤ x < 0.75), new reflections emerged, corresponding to P3-phase [13,14]. This phase coexisted with the initial O3-phase, which is in good harmony with the voltage plateau at (3.10–3.15) V. When Na extraction continued in the (0.40 ≤ x < 0.68) regions, this new P3-phase grew at the expense of the disappearing O3-phase. Further Na extraction in the (0.34 ≤ x < 0.40) regions led to the emergence of new reflections designated as O’3-phase (which resembles the pristine O3-phase) and coexisted with the P3-phase. The appearance of the new O’3-phase in the high voltage region suggests the shrinkage of the interlayer axis, rooted in insufficient Na ions in the Na-layer, which as more Na ions are extracted cannot support the structure. As Na extraction continued to x = 0.28 (Na_0.28_Fe_0.40_Co_0.40_Ti_0.20_O_2_, end of charge), the O’3-phase grew in strength, while the P3-phase disappeared. Therefore, during the first charge, the NFCTO electrode experienced O3 to P3 to O’3 phase transformations. It is worth noting that the interlayer spacing (i.e., O3 (003) peak shift around 16.5°) shows non-monotonic expansion and shrinkage during charging. In contrast, the intraplanar (‘ab’ plane) spacing (i.e., (110) peak shift around 64.5°) exhibits monotonic continuous shrinkage due to the TM’s oxidation, reflecting the straightforward correlation between TM’s oxidation state and intraplanar distance. 

In the reverse process (discharging), interestingly, asymmetric phase transition behavior was observed. Unlike the sequential phase transitions of O3 → P3 → O’3 during charge, only one phase transition from O’3 to the original O3 was observed during discharge. Upon Na insertion, the O’3 phase showed solid-solution behavior, and existed until the later stage of discharge (0.28 < x ≤ 0.69), followed by a phase transition to the original O3 phase at x = 0.69, which is evidenced by splitting of the O’3 (20-2) peak at ~37° into O3 (101) and (102) peaks. When Na insertion was 0.39 < x ≤ 0.69, the (001) reflection of the O’3-phase slowly shifted to lower 2θ values, expanding the c axis due to Na insertion; from this point to the end of discharge (x = 0.84), O3 (003) peak continued to shift to higher 2θ values. We note that the (003) reflection of the O3 phase corresponds to the (001) reflection of the O’3 phase originating from the monoclinic distortion. This O3 (003) peak shift towards higher 2θ values is ascribed to the extra Na insertion into the available vacant sites (20% vacancies). The additional insertion of Na^+^ ions reduces the repulsion between the oxide layers, thereby decreasing the interlayer distance (lattice parameter along the ‘c’ axis). Unlike complex (003) peak shift, the (110) peak, corresponding to the intraplanar distance along the ‘ab’ plane, again shows a monotonic and continuous shift toward lower angles (i.e., expansion) during discharge due to the continuous reduction of Fe^4+^ and Co^4+^.

Summarizing the in situ XRD result, the NFCTO electrode experienced O3 → P3 → O’3 phase transformations during charging, which is restored to the original O3-phase directly from O’3, but with excess Na ion insertion after discharging. This phase transition behavior is further supported by the dQ/dV profiles shown in Figure 2a. Close inspection of the dQ/dV curves suggests that during the first charging, the NFCTO has to undergo two different phase transformations, corresponding to P3 and O’3. However, during discharging, only one peak at 2.9 V (corresponding to ~ Na0.7) is observed, which is attributable to the O’3 → O3 phase transition. In the subsequent CV cycles, the electrode undergoes only one structural phase transition between the O3 and O’3 phases. This single-phase transformation during further cycling warrants the NFCTO as a highly stable electrode material. This result coincides well with a slightly higher first discharge capacity than the charge capacity and asymmetric voltage profiles during the first cycle (Figure 2). We note that such reversible but asymmetric phase transition behavior has not been observed in previously reported Na-deficient O3-type Na_0.9_[Cu_0.22_Fe_0.30_Mn_0.48_]O_2_ and overcharged O3-type Na(Mn_0.25_Fe_0.25_Co_0.25_Ni_0.25_)O_2_ cathode materials [13,14]. 

### 3.4. X-ray Absorption Spectroscopy (XAS) Analysis

**Figure 4:**
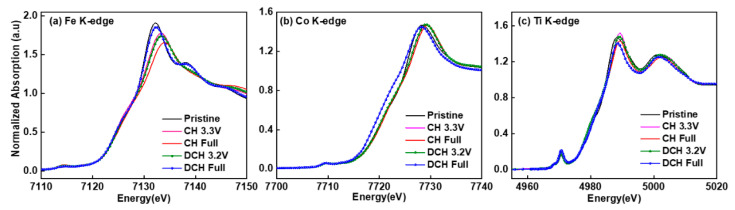
Normalized ex situ XANES spectra of the NFCTO electrode at (**a**) Fe, (**b**) Co and (**c**) Ti K-edges at various charge and discharge states.

The charge compensation mechanism during (de)sodiation was further investigated by ex-situ X-ray absorption spectroscopy (XAS) measured at Fe, Co, and Ti K-edges. The X-ray absorption near edge structure (XANES) spectra provide a straightforward and effective tool to reveal the average oxidation state of transition metals by comparing the edge energy positions. Figure S3 of the SI compares the XANES spectra of the pristine NFCTO electrode with reference compounds having known oxidation states (e.g., Fe(II)O, Fe(III)_2_O_3_, Co(II)O, LiCo(III)O_2_, and Li_4_Ti(IV)_5_O_12_), confirming the oxidation states of Fe^3+^, Co^3+^, and Ti^4+^ in the pristine NFCTO electrode. Figure 4a–c demonstrates the Fe, Co, and Ti K-edge XANES spectra of the NFCTO electrodes at various states of (dis)charge in the first cycle. 

The Fe spectra showed a clear edge shift to higher energy after full charge (4.0V) and fully returned to the original edge position after full discharge (2.0V), demonstrating quite reversible Fe^3+/4+^ charge compensation reaction during the 1st cycle. It is worth noting that such reversible Fe^3+/4+^ redox reaction probed by Fe K-edge spectra is in stark contrast to the pure oxygen redox reaction excluding Fe^3+/4+^ redox for the charge compensation of O3-NaFeO_2_ analogue during the 1st cycle [15]. We assume that substituting the sole Fe^3+^ by Co^3+^ and Ti^4+^ in layer-structured O3 stacking could activate the reversible Fe^3+/4+^ redox reaction, which requires further investigation. On the other hand, only a slight edge shift to higher energy was observed in the Co spectra after full charge (4.0V), suggesting partial oxidation of Co above 3+. Interestingly, during discharging, the Co spectra shifted noticeably below the original energy position after full discharge (2.0V), implying a partial reduction to 2+. This unexpected behavior is assigned to the extra Na ions inserted into the NFCTO material due to its Na deficiencies, which is responsible for the extra capacity observed during the 1st discharge. In contrast to Fe and Co spectra, Ti spectra exhibited no appreciable energy shift, but with only minor intensity changes during charging and discharge, which can be assigned to local-structure environment changes around Ti ions, as suggested by previous reports [12,16,17]. 

**Figure 5:**
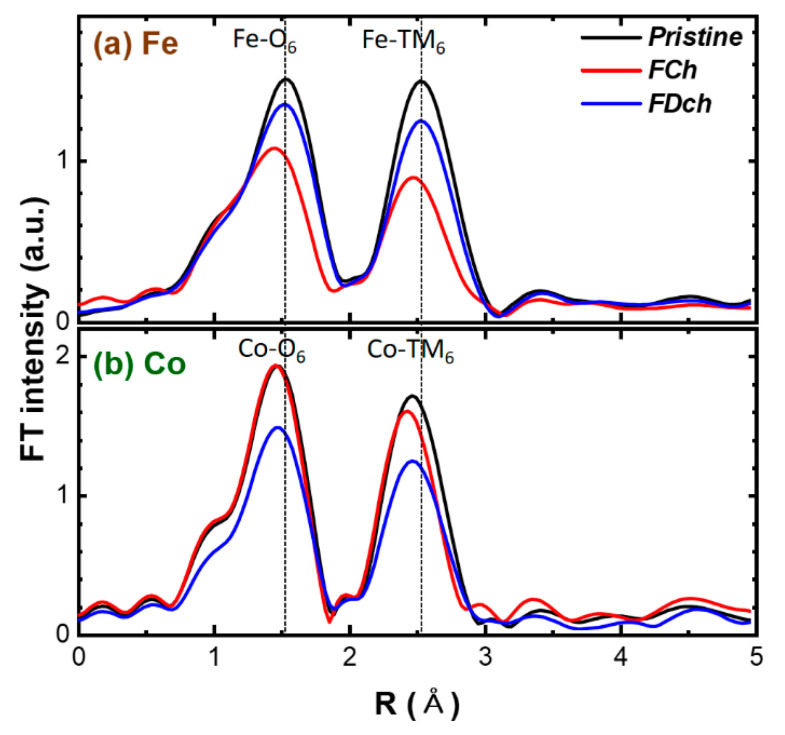
Ex situ EXAFS spectra at (**a**) Fe and (**b**) Co K-edges of NFCTO electrode at pristine, fully charged (4.0V) and fully discharged (2.0V) sates.

We further employed the Fe and Co K-edge EXAFS analysis to verify the charge compensation mechanism proposed by the XANES analysis. Note that the Ti K-edge EXAFS was excluded as Ti^4+^ remains unchanged during charge and discharge. The Fourier-transformed (FT)-EXAFS spectra (not phase-corrected FT, causing shorter bond lengths in the plots than for the real ones by ~0.3–0.4 Å) at Fe and Co Ti K-edges are shown in Figure 5. Both Fe and Co K-edge EXAFS spectra for the pristine electrode show very similar features with two distinct peaks; the first peaks around 1.5 Å for the nearest TM-O_6_ bond and the second peaks at around 2.5 Å for the second nearest TM-TM_6_ bond within the TM layers. This result confirms the formation of the layer-structured phase, where Fe and Co atoms are randomly distributed in the TM sites. The EXAFS fitting analysis provides quantitative (or structural) information from the best-fits about the TM-O bond length changes during charge and discharge (Supplementary Materials Table S2 in SI). The experimental and best-fits of the Fe and Co K-edge FT-EXAFS spectra and corresponding quantitative structural parameters are shown in Supplementary Materials Figure S4 and Supplementary Materials Table S2 in SI. 

The bond length for the first neighboring transition metal and oxygen is very sensitive to the valance changes. Hence, the first TM-O peak position in the EXAFS spectra is a good indicator of the oxidation state change during charging and discharging. The Fe-O bond length reversibly decreased (2.02 → 1.94 Å) and increased (1.94 → 2.01 Å) during charge and discharge, confirming the reversible Fe3+/4+ redox reactions. On the other hand, the Co-O bond length slightly decreased (1.93 → 1.91 Å) during charge and increased (1.91 → 1.94 Å) after 1st discharge, revealing partial redox activity of Co3+/4+ which agrees well with the XANES result. It is worth noting that the overall intensity of Fe/Co-O_6_ and Fe/Co-TM_6_ peaks notably decreased (i.e., increased Debye–Waller factor, σ^2^, in Supplementary Materials Table S2 in SI) after the charge, suggesting local structural distortion at the charged state.

Therefore, based on the XANES and EXAFS results, in the first cycle, Fe undergoes reversible 3+/4+ redox reaction, Co changes partially from 3+ to 4+ (during charging) and partially from 4+ to 2+ (during discharging), while Ti does not contribute to the charge compensation reaction. We attribute the prevention of structural degradation during sodium insertion and extraction during cycling to the stable Ti in the layered NFTCO structure, giving rise to the high capacity retention for 50 cycles. Comparison with the performance of the other reported O3-type layered cathode materials summarized in Supplementary Materials Table S3 of the SI shows that the NFCTO electrode offers competitive electrochemical performance.

## 4. Conclusions

In summary, we successfully designed and prepared a new O3-type layered Na_0.80_[Fe_0.40_Co_0.40_Ti_0.20_]O_2_(NFCTO) cathode material using a double-substitution concept of Co in the parent NaFe_0.5_Co_0.5_O_2_, having the general formula Na_1-x_[Fe_0.5-x/2_Co_0.5-x/2_M^4+^_x_]O_2_ (M^4+^ = tetravalent ions). The material delivered a high first charge and discharge capacity of 106 and 108 mAhg^−1^ at 0.1C at (2.0–4.0V), showing slightly higher discharge capacity due to excess Na^+^ insertion, which stabilized at ~110 mAhg^−1^ in the first few cycles and reached ~87 mAhg^−1^ after 50 cycles. Notably, the first charge–discharge profile showed asymmetric features, two-voltage plateaus during charging, and a sloping and single-voltage plateau during discharging. This asymmetric yet reversible redox reaction is attributed to asymmetric phase transitions, O3 → P3 → O’3 phase during charging, restored to the original O3-phase directly from O’3 during discharging (O’3 → O3), which was identified by the synchrotron in situ XRD results. Ex situ XANES analysis reveals a quite reversible Fe^4+^/Fe^3+^ redox reaction, while Ti^4+^ remains unchanged during the 1st cycle. Interestingly, Co reveals asymmetric redox behavior, showing partial oxidation from 3+ to 4+ (after1st charge), followed by a reduction from 4+ to below the pristine 3+ state (after 1st discharge), which is responsible for the asymmetric redox reaction. We demonstrated that O3-type layered cathodes with the general formula Na_1-x_[Fe_0.5-x/2_Co_0.5-x/2_M^4+^_x_] O_2_ (M^4+^ = tetravalent ions) can provide an exciting opportunity to explore the interplay between rich-chemistry and sodium storage properties, which may lead to the development of new cathode materials for SIBs.

## Data Availability

The article and supplementary materials contain all data.

## Supplementary Materials

The following are available online at https://www.mdpi.com/article/10.3390/ma14092363/s1, Supplementary Materials Figure S1: XRD patterns of NFCTO sample prepared at 800, 900, and 1000 °C; Supplementary Materials Figure S2: EDS spectrum of NFCTO sample; Supplementary Materials Figure S3: Normalized ex-situ XANES spectra at (a) Fe, (b) Co, and (c) Ti K-edges of NFCTO pristine electrode, compared to the known reference materials; Figure S4: Ex situ EXAFS spectra at (a) Fe and (b) Co K-edges of NFCTO electrode at pristine, fully charged (4.0V) and fully discharged (2.0V) sates. Least-square fits for the calculated FT-EXAFS phase are shown in yellow color-coded lines while experimental data are shown in filled circles. The FT magnitude of the EXAFS spectra have not been corrected for phase shift; Supplementary Materials Table S1: Crystallographic parameters and atomic coordinates of the Na_0.8_Fe_0.4_Co_0.4_Ti_0.2_O_4_ pristine sample $\left(R\bar{3}m\;\mathrm{space}\;\mathrm{group}\right)$, obtained after Rietveld refinement of synchrotron data; Table S2: Structural parameters obtained from best-fit results of Fe and Co K-edge EXAFS spectra. CN: Coordination number; ΔE: inner shell potential shift; r: bond length; σ^2^: Debye–Waller factor; R: EXAFS R-factor; χ_v_^2^: reduced chi-squared; Supplementary Materials Table S3: Comparison of the NFCTO electrode with other reported layer-structured electrode materials.
