# Supplementary material for: New O3-Type Layer-Structured Na0.80[Fe0.40Co0.40Ti0.20]O2 Cathode Material for Rechargeable Sodium-Ion Batteries
Source: Materials (Basel). 2021 May 1;14(9):2363. doi: 10.3390/ma14092363 (PMC8124707; doi:10.3390/ma14092363)
Supplement: Supplementary file 1 [file materials-14-02363-s001.zip › materials-1189649-supplementary.pdf]

materials

MDPI

Supplementary Materials

New O3-type layer-structured $Na_{0.80}[Fe_{0.40}Co_{0.40}Ti_{0.20}]O_2$cathode material for Rechargeable Sodium-ion batteries

Daniel A. Anang[1,2], Deu S. Bhange[3], Basit Ali[1] and Kyung-Wan Nam[1,*]

[1] Department of Energy and Materials Engineering, Dongguk University-Seoul 04620, Seoul, Republic of Korea
[2] Department of Chemical Engineering, Kwame Nkrumah University of Science and Technology, PMB, Kumasi, Ghana
[3] Department of Chemistry, Shivaji University, Kolhapur, 416004, India
* Correspondence: knam@dongguk.edu; Tel: +82-2-2290-4978;Fax: +82-2-2268-8550

Citation: Anang, D.A.; Bhange, D.S.; Ali, B.; Nam, K.-W. New O3-Type Layer-Structured $Na_{0.80}[Fe_{0.40}Co_{0.40}Ti_{0.20}]O_2$ Cathode Material for Rechargeable Sodium-Ion Batteries. *Materials* **2021**, *14*, 2363. https://doi.org/10.3390/ma14092363

Received: 2 April 2021
Accepted: 29 April 2021
Published: date

Publisher's Note: MDPI stays neutral with regard to jurisdictional claims in published maps and institutional affiliations.

Copyright: © 2021 by the authors. Submitted for possible open access publication under the terms and conditions of the Creative Commons Attribution (CC BY) license (http://creativecommons.org/licenses/by/4.0/).

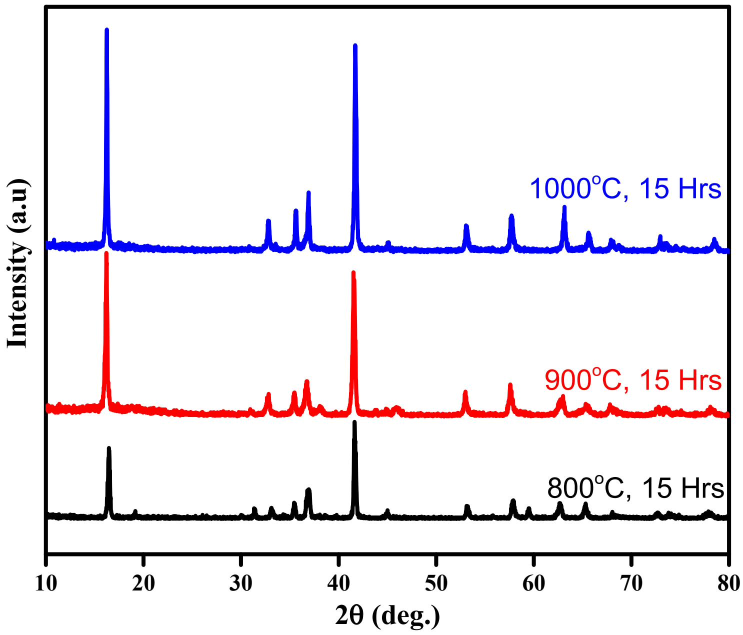

Figure S1. XRD patterns of NFCTO sample prepared at 800 °C, 900 °C and 1000 °C.

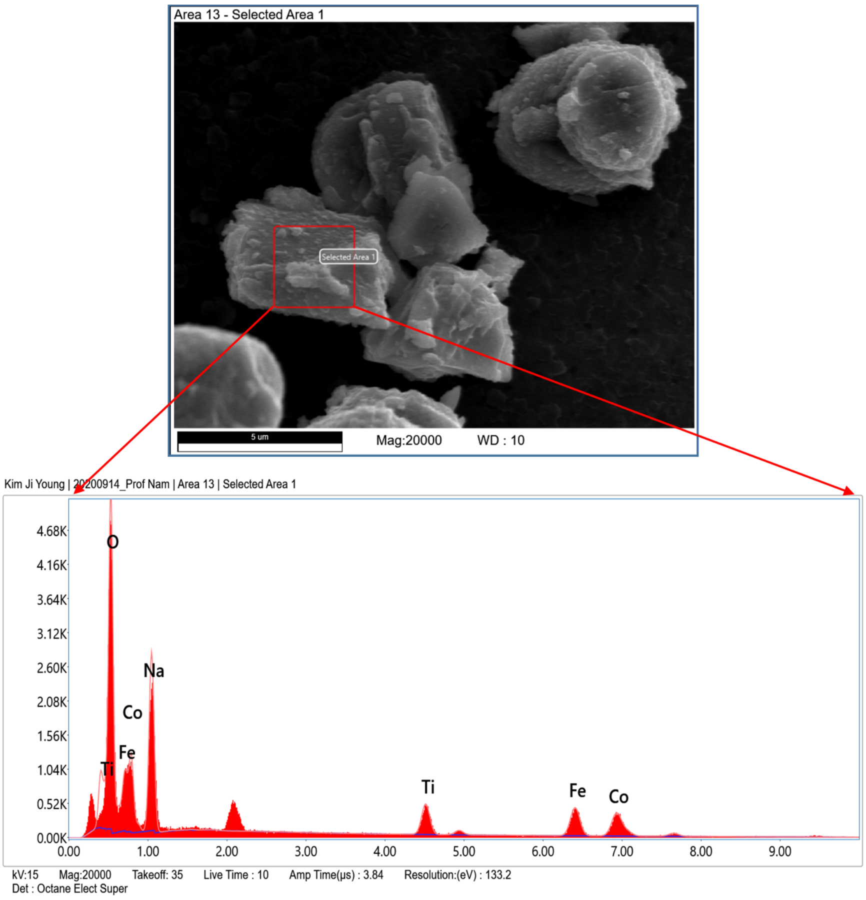

Figure S2. EDS spectrum of NFCTO sample.

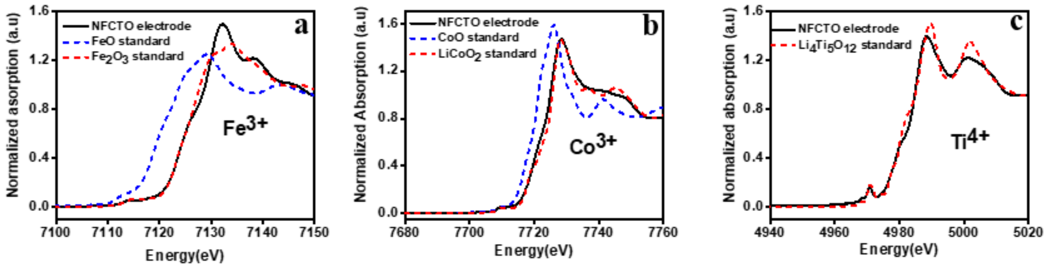

Figure S3. Normalized ex-situ XANES spectra at (**a**) Fe (**b**) Co and (**c**) Ti K-edges of NFCTO pristine electrode compared to known reference materials.

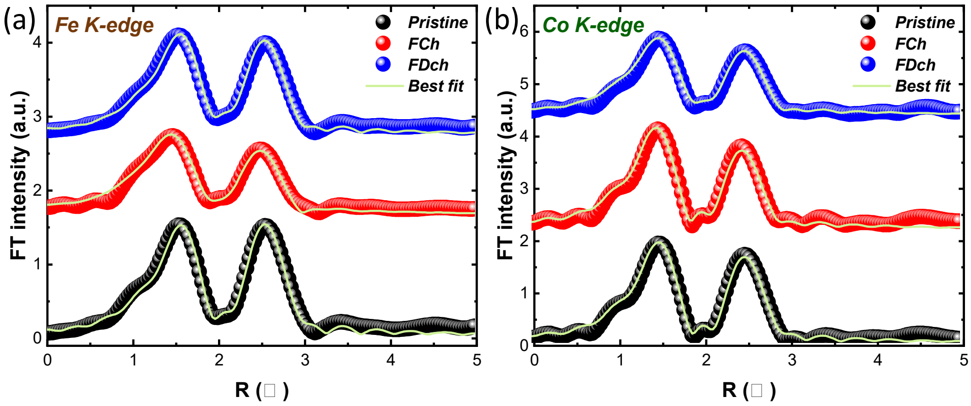

Figure S4. *Ex situ* EXAFS spectra at (**a**) Fe and (**b**) Co K-edges of NFCTO electrode at pristine, fully charged (4.0V) and fully discharged (2.0V) sates. Least-square fits for the calculated FT-EXAFS phase are shown in yellow color-coded lines while experimental data are shown in filled circles. The FT magnitude of the EXAFS spectra have not been corrected for phase shift.

Table S1. Crystallographic parameters and atomic coordinates of the $Na_{0.8}Fe_{0.4}Co_{0.4}Ti_{0.2}O_4$pristine sample ($R\bar{3}m$ space group),obtained after Rietveld refinement of synchrotron data.

Atom	Multiplicity	*x*	*y*	*z*	Occupancy	Uiso
Na	3	0.0000	0.0000	0.0000	0.8	0.0285 (9)
Fe1	3	0.0000	0.0000	0.5000	0.4	0.0114 (4)
Co1	3	0.0000	0.0000	0.5000	0.4	0.0114 (4)
Ti1	3	0.0000	0.0000	0.5000	0.2	0.0114 (4)
O1	6	0.0000	0.0000	0.2305 (13)	1.0	0.0051 (7)

Space group: $R\bar{3}m$, a=2.9475 (1) Å, b =2.9475 (1) Å, c=16.3621(2) Å, V=123.10 (2) Å^3.
Agreement parameters: Rwp=11.56%, Rp=8.86%, R_{F^2} = 7.07%.

Table S2. Structural parameters obtained from best-fit results of Fe and Co K-edge EXAFS spectra. CN: Coordination number; ΔE: inner shell potential shift; r: bond length; σ^2: Debye-Waller factor; R: EXAFS R-factor; χ_v^2: Reduced chi-squared.

TM	state	Path	CN	ΔE (eV)	r (Å)	$\sigma^2\times10^{-3}$ (Å^2)	R	χ_v^2
Fe	Pristine	Fe-O	6	1.59±0.58	2.020±0.006	5.8±0.6	0.004	534
		Fe-M	6		2.956±0.005	6.1±0.4		
	Charge	Fe-O	6	−1.02±0.80	1.939±0.007	11±0.7	0.006	202
		Fe-M	6		2.924±0.008	12±0.7		
	Discharge	Fe-O	6	1.42±0.53	2.012±0.005	7.1±0.5	0.003	97
		Fe-M	6		2.959±0.005	7.9±0.4		
Co	Pristine	Co-O	6	−0.27±0.98	1.930±0.008	4.4±0.7	0.009	575
		Co-M	6		2.889±0.008	6.5±0.6		
	Charge	Co-O	6	−1.64±1.30	1.906±0.010	4.4±0.9	0.015	792
		Co-M	6		2.866±0.011	7.6±0.9		
	Discharge	Co-O	6	−0.53±1.28	1.942±0.011	7.6±1.0	0.015	549
		Co-M	6		2.890±0.012	9.3±0.9		

Table S3. Comparison of the NFCTO electrode with other reported layer-structured electrode materials.

Electrode Material	First discharge capacity ($mAhg^{-1}$)	Capacity retention	Rate performance	Reference
α-$NaFeO_2$	80 at 12.1 mAg^{-1}	75% after 30 cycles	_	[1]
$NaFe_{0.5}Co_{0.5}O_2$	160 at 12 mAg^{-1}	85% after 5 cycles	30C (105 $mAhg^{-1}$)	[2]
P2-$Na_{2/3}Mn_{1/2}Co_{1/2}O_2$	123 at 30 mAg^{-1}	69% after 30 cycles	_	[3]
$Na[CuFeMnTi]_{1/4}O_2$	94 at 10 mAg^{-1}	60% after 90 cycles	_	[4]
$NaFe_{0.45}Co_{0.45}Ti_{0.1}O_2$	144 at 0.1C	70% after 50 cycles	_	[5]
Na_2FePO_4F	110 at 6.2 mAg^{-1}	75% after 20 cycles	4C (30 $mAhg^{-1}$)	[6]
$Na_2MnP_2O_7$	80 at 0.05C	82% after 15 cycles	_	[7]
$NaNi_{0.5}Mn_{0.47}Sn_{0.03}O_2$	191 at 0.1C	85% after 100 cycles	8C (56 $mAhg^{-1}$)	[8]
$Na[NiCoFeTi]_{1/4}O_2$	116 at 12 mAg^{-1}	93% after 100 cycles	20C (94 $mAhg^{-1}$)	[9]
$Na[NiCoFeMn]_{1/4}O_2$	180 at 0.1 C	_	_	[10]
$Na_{0.6}Cr_{0.6}Ti_{0.4}O_2$	180 at 0.1 C	94% after 200 cycles	2C (61 $mAhg^{-1}$)	[11]
$Na_3Ni_2SbO_6$	117 at 0.1C	95% after 50 cycles	30C (90 $mAhg^{-1}$)	[12]
$NaMn_2O_4$	65 at 5 mAg^{-1}	94% after 200 cycles	_	[13]
**NFCTO**	**108 at 0.1C**	**80% after 50 cycles**	**5C (57 $mAhg^{-1}$)**	This work

References

[1] N. Yabuuchi, H. Yoshida, S. Komaba, *Electrochemistry*, 80 (2012) 716-719.

[2] H. Yoshida, N. Yabuuchi, S. Komaba, *Electrochem Commun.* 34 (2013) 60–63.

[3] X. Wang, M. Tamaru, M. Okubo, A. Yamada, *J. Phys. Chem. C*. 117 (2013) 15545-15551.

[4] L. –Q. Mu, Y. –S. Hu, L. –Q. Chen, *Chinese Phys. B*. 24 (2015) 038202.

[5] M. Kouthaman, K. Kannan, P. Arjunan, T. Meenatchi, R. Subadevi, M. Sivakumar, *Materials Letter* 276 (2020) 128181.

[6] Y. Kawabe, N. Yabuuchi, M. Kajiyama, N. Fukuhara, T. Inamasu, R. Okuyama, I. Nakai, S. Komaba, *Electrochem. Commun*. 13 (2011) 1225-1228.

[7] P. Barpanda, T. Ye, M. Avdeev, S. –C. Chung, Yamada, *J. Mater. Chem. A*. 1 (2013) 4194.

[8] Y. Meng, J. An, L. Chen, G. Chen, L. Shi, M. Lu, D. Zhang, *Chem Commun.* 56(58) (2020) 8079-8082.

[9] J.-L. Yue, Y.-N. Zhou, X. Yu, S.-M. Bak, X.-Q. Yang, Z.-W. Fu, *J. Mater. Chem. A*.**,** 3 (2015) 23261–23267.

[10] X. Li, D. Wu, Y.-N. Zhou, L. Liu, X.-Q. Yang, G. Ceder, *Electrochem. Commun*. 49 (2014) 51–54.

[11] Y. Wang, R. Xiao, Y-S. Hu, M. Avdeev, L. Chen, *Nature Commun*. 6 (2015) 1–9.

[12] D. Yuan, X. Liang, L. Wu, Y. Cao, X. Ai, J. Feng, H. Yang, *Adv Mater*. 26 (2014) 6301-6306.

[13] X. Liu, X. Wang, A. Iyo, H. Yu, D. Lia, H. Zhou, *J. Mater. Chem. A*, 2 (2014) 14822.
